# Suppression potential of selected vermicomposts against root-knot nematode (*Meloidogyne incognita*) under *in vitro*, pot, and field conditions

**DOI:** 10.3389/fpls.2025.1532800

**Published:** 2025-03-11

**Authors:** Zerihun Getachew Gebrehana, Mesfin T. Gebremikael, Sheleme Beyene, Wim M. L. Wesemael, Stefaan De Neve

**Affiliations:** ^1^ Research Group Soil Fertility and Nutrient Management, Faculty of Bioscience Engineering, Ghent University, Ghent, Belgium; ^2^ Assosa Agricultural Research Center, Ethiopian Institute of Agriculture Research, Addis Ababa, Ethiopia; ^3^ Department of Food Science, Aarhus University, Aarhus, Denmark; ^4^ School of Plant and Horticultural Sciences, College of Agriculture, Hawassa University, Hawassa, Ethiopia; ^5^ Plant Sciences Unit—Crop Protection, Institute for Agricultural and Fisheries Research (ILVO), Merelbeke, Belgium; ^6^ Laboratory for Agrozoology, Faculty of Bioscience Engineering, Ghent University, Ghent, Belgium

**Keywords:** organic amendment, soil health, tomato production, biocontrol agent, *Meloidogyne* suppression

## Abstract

The root-knot nematode *Meloidogyne incognita* presents a serious threat to high-value crops in tropical and subtropical regions, particularly in Ethiopia, causing substantial yield and quality losses. Vermicompost, whether applied in solid form or as an extract, has shown promise in managing root-knot nematodes (RKNs). However, its effectiveness is influenced by factors such as the quality and type of vermicompost, the application rate, and the composition of parasitic nematode communities in the soil. This study utilized selected vermicomposts at varying rates in in vitro, pot, and field experiments to evaluate their potential for suppressing *M. incognita* and their effects on the growth and yield of tomato and hot pepper. The *in vitro* experiments demonstrated that all vermicompost extracts exhibited toxicity to J2. In particular, VC10 and VC11 showed higher efficacy, resulting in 55% and 78% mortality of J2 after 24 and 72 h of exposure, respectively, compared to the control and VC12. The interaction between vermicompost type, application rate, and nematode density significantly influenced tomato growth and nematode parameters in the pot experiment. The application of VC10 and VC11 at high doses (10 and 20 t ha^−1^) and low nematode density (50 J2) increased root fresh weight while reducing galls and nematode populations in tomato roots. Conversely, VC12 at a high application rate (20 t ha^−1^) and high nematode density (500 J2) led to an increase in root galls and nematode populations, suggesting a preference for RKNs rather than the expected nematicidal effect. The study indicates that the suppressive effect of vermicompost on nematodes varies with nematode density, depending on the type and amount of vermicompost used. Field experiments revealed that vermicompost amendments not only suppressed posttreatment nematode populations but also significantly improved hot pepper yield. Particularly, VC10 applied at high rates (10 and 20 t ha−1) resulted in lower nematode densities and higher marketable fruit yield compared to other vermicompost treatments and the conventional treatments (control, farmer practice, and recommended fertilizer). This highlights the long-term benefits of vermicompost application for nematode management and soil health. In addition, vermicompost amendments improved soil chemical properties. Overall, vermicompost offers greater benefits than farmers’ practices and high-cost chemical fertilizers for soil improvement, while also enhancing tomato and hot pepper yields in nematode-infested smallholder farms.

## 1 Introduction

Root-knot nematodes (RKNs) pose a significant threat to crops worldwide (Rao et al., 2017), leading to annual losses in the billions of dollars (Khan et al., 2019). *Meloidogyne* spp. is particularly notorious for its economic destructiveness, making it a major concern for agricultural productivity (Jones et al., 2013; Zuhair et al., 2022). Among all species, *Meloidogyne incognita* is considered the world’s most damaging crop pathogen (Janssen et al., 2016). Its prevalence is most prominent in tropical and subtropical regions (Ghareeb et al., 2022), where it inflicts significant yield losses ranging from 25% to 100% in vegetable crops (Seid et al., 2015). In Ethiopia, smallholder farmers suffer considerable losses, primarily due to the widespread occurrence of *M. incognita*, which leads to yield losses of up to 50% in vegetable-growing areas (tomatoes) (Seid et al., 2019). *Meloidogyne incognita* invades plant roots and feeds on their internal tissues after establishing a permanent feeding site (giant cells), causing damage to the root system and impairing the plant’s ability to absorb water and nutrients (Karssen et al., 2013). Despite the increasing concern over the severe damage caused by RKNs, nematode infestation is often underestimated in Ethiopia. This is primarily due to the nature of the damage caused by nematodes and the limited awareness of effective management approaches (Meressa, 2014; Abebe et al., 2015). The pathogenicity of RKNs remains a significant challenge, largely because smallholder farmers have a limited understanding of nematodes and a lack of expertise in managing infestations. This knowledge gap exacerbates the problem, as farmers often struggle to identify or implement effective control measures, allowing RKN populations to persist and cause substantial crop losses.

To mitigate the effects of RKNs, various management approaches have been explored, including the use of chemical nematicides (Rao et al., 2017; Kaur et al., 2022). However, concerns about environmental safety, human health, and the sustainability of agricultural practices have led to a shift toward biological control methods, particularly the application of organic amendments (Oka, 2010; Thoden et al., 2011). Vermicompost, produced through the decomposition of organic waste by the synergistic activity of earthworms and microorganisms, has emerged as a promising alternative (Dominguez et al., 2019). Its unique characteristics—nutrient richness, microbial activity, and biological control properties—make vermicompost an effective tool for enhancing soil and plant health while suppressing plant diseases and pests (Lazcano and Domínguez, 2011; Pathma and Sakthivel, 2012).

Studies have demonstrated that vermicompost effectively reduces plant-parasitic nematode infestations in various horticultural crops, including tomatoes, hot peppers, grapes, strawberries, and carrots (Simsek-Ersahin, 2011; Joshi et al., 2015; Rao et al., 2017). Its application can increase the population of beneficial nematodes, such as bacterial or fungal predators, while simultaneously reducing or suppressing economically significant plant-parasitic nematodes (Renčo, 2013). Furthermore, vermicompost promotes root defenses against RKNs by promoting the accumulation of defense compounds or modifying soil properties (Xiao et al., 2016).

Recently, there has been a growing interest in utilizing vermicompost extract/tea as an effective method alongside solid vermicompost for various agricultural applications (Tikoria et al., 2022). Vermicompost extract is a liquid product obtained by soaking harvested vermicompost in water, which facilitates the extraction of soluble nutrients and beneficial microbes (Arancon et al., 2012). Several studies have demonstrated the efficacy of vermicompost extract in suppressing RKNs (Arancon et al., 2007; Edwards et al., 2007; Yatoo et al., 2021). Both solid vermicompost and its liquid extract form are highly suitable (Sulaiman and Mohamad, 2020) and hold great potential for crop production and suppression of *M. incognita* (Kaur et al., 2022).

Despite the significant damage caused by *M. incognita*, a species confirmed as the dominant RKNs in Ethiopian vegetable farms through morphological and molecular studies (Seid et al., 2019), management options remain limited. Furthermore, the suppressive effects of vermicompost and other organic sources are well-documented in different countries; its efficacy can vary depending on the source, type, and application method of the vermicompost, the nematode species, and population density. Notably, there is a lack of comprehensive studies evaluating the use of different vermicompost types under varying experimental conditions. To address this gap, the present study investigated the suppressive potential of selected vermicomposts against *M. incognita* across *in vitro*, pot, and field experiments. The study focused on understanding the interactions between vermicompost type, application rate, nematode density, and their combined effects on nematode suppression and crop growth. By establishing relationships between controlled and field conditions, the study aimed to provide insights into the broader applicability of vermicompost for managing RKNs in nematode-infested smallholder farms. Furthermore, by identifying the suppressive effects of vermicomposts, the study offers insights into the nematode populations posttreatment, a critical but underexplored area in nematode management. The results could be used for soil and plant health improvement.

## 2 Materials and methods

### 2.1 Vermicomposts used

The previous vermicomposting experiment (Gebrehana et al., 2023) utilized a variety of mixed substrates composed of cow manure (CM) and donkey manure (DM), along with three crop residue combinations (50% w/w): maize and soybean (MS), maize and banana (MB), and soybean and banana (SB). The final substrate mix in each experimental box was prepared by blending 2.1 kg (dry weight) of CM or DM with 0.45 kg of each of the two selected crop residues (MS, MB, or SB). Three earthworm species, *Eudrilus eugenia* (EU), *Eisenia fetida* (EF), and *Eisenia andrei* (EA), were independently introduced to the substrates. The study employed a factorial experimental design comprising two types of manure, three crop residue combinations, and three earthworm species, resulting in 18 treatment combinations. The species of earthworms used in the vermicomposting process were previously identified using taxonomic characteristics specific to the genus and species (Blakemore, 2015; Domínguez, 2018). In addition, a control treatment with undecomposed substrates (without earthworm inoculation) was included for comparison. Five vermicomposts were selected from these treatments based on their biochemical properties, including total carbon, nutrient concentrations (N, P, and S), and C:N ratios (Gebrehana et al., 2023). These selections were further informed by microbial characteristics and their suitability for promoting earthworm growth. This approach provided a comprehensive assessment of substrate performance and earthworm compatibility.

The selected vermicomposts were as follows: VC10, produced from cow manure, soybean, and banana residues using *Eudrilus eugeniae*; VC11, produced from cow manure, soybean, and banana residues using *Eisenia Andrei*; and VC12, produced from cow manure, soybean, and banana residues using *Eisenia fetida*. Vermicompost extract/tea was prepared from each vermicompost by soaking 100 g of vermicompost in 500 ml of water (1:5 ratio), mixing using a centrifuge, and aerating. Each vermicompost extract was filtered using a kitchen strainer to separate solids from liquids prior to application for the *in vitro* experiment.

### 2.2 Nematode inoculum for *in vitro* and pot experiments

The *Meloidogyne incognita* population used in this study was originally obtained from tomato-growing regions in Ethiopia. Morphological and molecular characterization of individual egg masses extracted from infected tomato plants cultivated under controlled pot conditions at ILVO, Belgium, was conducted as described by Seid et al. (2019). Before initiating the present experiment, the *M. incognita* population was collected and identified from tomato-growing fields in Ethiopia using molecular techniques, including DNA and isozyme-based methods, as detailed by Seid et al. (2019). To ensure accuracy, species identification and differentiation were conducted using both morphological taxonomic features and molecular criteria before the nematodes were prepared for multiplication. The nematodes were subsequently multiplied on susceptible tomato plants (*Solanum lycopersicum* L., variety Marmande), cultivated in 5-kg pots filled with silver sand for 10–12 weeks. The inoculum was prepared in the nematology laboratory at ILVO from heavily galled tomato roots infected by *M. incognita*. Then, to obtain the required second-stage juveniles (J2), the galled tomato roots were chopped into 1–2 cm pieces and processed in a spray-mist chamber. The steps for nematode extraction and preparation were as follows:

Baermann pan extraction: The root sections were placed on a Baermann pan setup, facilitating the collection of freshly hatched J2. This method relies on the active movement of juveniles from root fragments into water (Hooper et al., 2005).

Centrifugation parameters: The extract containing eggs and nematodes was centrifuged at 1,500 rpm for 5 min to separate eggs and debris. This speed is optimal to prevent damage to nematodes while effectively concentrating them. The nematode extract was diluted with sterile distilled water to ensure the J2 remained uncontaminated by external microorganisms. Sterile water also minimizes the risk of bacterial or fungal contamination during experiments. Freshly hatched J2 were collected every 24 h, and the water in the dish was replenished with fresh sterile tap water. J2 were collected for three consecutive days and stored at room temperature in sterile distilled water until use. The density of J2 in the extract was determined using a stereomicroscope. An aliquot of the suspension was taken, and J2 was counted manually using a nematode counting dish. The calculated population was used to standardize inoculum density both under *in vitro* and greenhouse experiments.2.

### 2.3 *In vitro* J2 mortality test

A volume of 3 ml of the cell-free culture filtrates from each vermicompost extract was placed in small glass dishes, measuring 20 mm in diameter and 10 mm in depth. Approximately 100 J2s were transferred to the glass dishes. The treatments (extracts from VC10, VC11, and VC12, and the control) were evaluated for mortality with five replicates in a completely randomized design. Using a stereomicroscope (Olympus, Tokyo, Japan ), the mortality of J2 was recorded separately after 24 and 72 h of exposure. Immobile J2 (paralyzed) were considered dead if they did not move when pricked with a fine needle. Mortality (%) was determined by dividing the number of dead J2 by the total number of J2.

### 2.4 Pot experiment

The purpose of this experiment was to determine if the addition of vermicompost and vermicompost quantities to a potting soil mix would suppress root gall formation and populations of *M. incognita* inoculated at different densities on tomato plants. The pot experiment included 36 factorially combined treatments, consisting of three types of vermicompost (VC10, VC11, and VC12), four vermicompost rates (0, 5, 10, and 20 t ha^−1^), and three densities of *M. incognita*—uninoculated, low (50 J2 per 100 cm^3^ of soil) or high (500 J2 per 100 cm^3^ of soil)—with each treatment replicated three times (**Table 1**). A total of 90 pots were used since the vermicompost application rate of 0 t ha^−1^ was the same across all vermicompost types. The experimental sterile soil, Nitisols, was thoroughly mixed with the different vermicompost types and placed in 1 L pots. The tomato seedlings of the susceptible cultivar var. Marmande were raised separately in a plastic tray (54 cm × 28 cm × 6 cm). Each plot received a single, 6-week-old tomato seedling var. Marmande, which was watered as needed. One week after transplanting, tomato seedlings were inoculated by pouring low (50 J2 per 100 cm^3^ of soil) and high (500 J2 per 100 cm^3^ of soil) densities of *M. incognita* around the root of the tomato plant. The suspensions of all individual densities were prepared from a single stock suspension. For inoculation, needles were uniformly inserted into the pots at equal distances from each other and 3–4 cm from the base. A 3-ml pipette containing the required amount of J2 was used to transfer the nematodes into the needles, and the needle content was gently released while the needles were slowly pulled up. This method ensured a random distribution of J2 along the vertical profile of the soil (Kefelegn et al., 2024). The control treatment consisted of plants treated similarly to the inoculated ones but without J2. Tomato plants were then maintained in a greenhouse under a 16-h light period at 22°C–24°C and an 8-h dark period at 18°C–20°C.

**Table 1:** Treatment details for pot and field experiments.

Pot experiment			Field experiment	
Vermicompost type	Vermicompost rate (t ha^−1^)	Nematode density (J2 per 100 cm^3^ of soil)	Fertilizer source (rate)	Equivalence amount of N applied (kg N ha^−1^)
VC10	0	Uninoculated	Control	0
VC11	5	Low (50 J2)	VC10 at 5 t ha^−1^	~ 60
VC12	10	High (500 J2)	VC10 at 10 t ha^−1^	120
**(3)**	20	**(3)**	VC10 at 20 t ha^−1^	240
	**(4)**		VC11 at 5 t ha^−1^	~ 55
3 * 4 * 3 = 36 treatment combinations			VC11 at 10 t ha^−1^	110
			VC11 at 20 t ha^−1^	220
			VC12 at 5 t ha^−1^	~ 55
			VC12 at 10 t ha^−1^	110
			VC12 at 20 t ha^−1^	220
			Farmers practice (FP)	Urea (23 kg N ha^−1^), cow manure (3 t ha^−1^)
			Recommended fertilizer (RF)	110 kg N ha^−1^ and 23 kg P ha^−1^

After 2 months, individual plants were cut at the soil level, and the roots were washed free of soil. The severity of root galls on tomato plants infected with *M. incognita* was scored using a 0–5 rating scale according to Quesenberry et al. (1989), where 0 = no galls, 1 = 1–2 galls, 2 = 3–10, 3 = 11–30, 4 = 31–100, and 5 > 100 galls per root system. The severity of nematode galls in the control treatments was not scored because no galls developed in these roots. J2 were extracted from the soil and the roots. The soil from each pot was thoroughly mixed, and a 100 cm^3^ subsample was taken. The roots were macerated using a commercial waring blender to liberate the nematodes from the root tissue. J2 were extracted separately from the soil and roots using the Hendrickx automated zonal centrifuge at ILVO, Merelbeke, Belgium (Hendrickx, 1995). The extracted J2 were counted using a binocular microscope, and the counts were used to calculate the final J2 population densities in both the root and soil.

### 2.5 Field experiment

The experimental field was located in western Ethiopia, about 670 km west of Addis Ababa. The study area is situated between 9°56′21.6″N and 034°39′44.5″E in western Ethiopia, has an altitude range of 1,462–1,580 m asl. The mean monthly temperature ranges from 15°C to 28°C, with an annual rainfall of 1,183 mm. The Assosa district has a mono-modal rainfall pattern, with a single rainy season. The soil is classified as Nitisols. The field experiment was conducted from February 2023 to the first of June 2023 using hot pepper (*Capsicum annuum* L.) seedlings grown at a farmer’s nursery site. The field was selected due to *Meloidgyne* spp. infestation detected through preliminary soil sampling. Tomato and hot pepper were previously grown in the field, where *Meloidogyne* spp. infection was confirmed. The experimental plots measured 2.4 m × 2.1 m, each containing four rows with seven plants per row. Twelve treatments were applied with the same vermicompost and rate as in the pot experiment. The recommended rate of fertilizers (RF) and farmers’ practice (FP) were also included as treatments in the field trial (**Table 1**), which used furrow irrigation. An unamended/fertilized control, with no amendments applied, was included for comparison. A chemical NPS fertilizer (19% N, 38% P, and 7% S) was applied at 100 kg ha^−1^ as a basal treatment to each plot. Urea was used as a treatment fertilizer (for 11 and 12, **Table 1**). The experiment followed a randomized complete block design (RCBD) with three replications. Root gall scores were recorded similarly to the pot experiment, and disease incidence (%) was calculated as the number of infected plants divided by the total plants per plot. Agronomic data, including plant height, fruit count (pods), fruit width and length, and marketable and nonmarketable yields, were recorded. Postharvest chemical analysis of field soil was also conducted.

#### 2.5.1 Soil chemical analyses

For the field experiment performed in 2023, three replicate surface soil samples (0–20 cm) were combined into one composite sample for chemical analysis (**Table 2**). Soil samples were also collected before planting and after harvesting to quantify the J2 nematode population. These samples were processed using the modified Baermann funnel technique, an efficient and widely used method for isolating nematodes from soil. This approach ensured accurate and consistent evaluation of nematode population dynamics throughout the experiment. Particle size distribution was determined using the Bouyoucos hydrometer method (Bouyoucos, 1962). Soil pH was measured in a 1:2.5 soil-to-water suspension using a pH meter. The Walkley–Black method was used to determine soil organic carbon (OC) content (Walkley and Black, 1934). Total N content was determined using the micro-Kjeldahl method, involving digestion, distillation, and titration procedures, as described by Nelson and Sommers, (1982). Available P was determined by the Bray II method (Bray and Kurtz, 1945). Cation exchange capacity (CEC) was determined by leaching the soil with 1N ammonium acetate (pH 7) (Van Reeuwijk, 2002), and available K was measured using a flame photometer. Available S was determined using the turbidimetric barium sulfate precipitation method (Getachew et al., 2017).

**Table 2:** Effects of vermicompost type, application rate, and nematode density on root fresh weight, gall score, root nematode population (*M. incognita* per root system), and soil nematode population (*M. incognita* per 100 cm^3^ of soil) under pot conditions.

Treatment	Root fresh weight (g)	Gall score (1 to 5)	*M. incognita* population	
			Root (egg, J2)	Soil (J2)
Type of vermicompost (VC)				
Unamended (0)	7.50 b	3.00 a	9,012.3 a	633.33 a
Vermicompost-10 (VC10)	14.58 a	2.22 c	4,919.2 b	128.89 b
Vermicompost-11 (VC11)	14.78 a	2.37 c	6,779.6 ab	111.11 b
Vermicompost-12 (VC12)	14.54 a	2.70 b	6,871.4 ab	197.78 b
LSD (Tukey’s test)	3.32	0.272	2,985.9	171.56
Vermicompost rate (VCR)				
Control (0)	7.50 c	3.00	9,012.3	633.33
5 t ha^−1^	10.57 bc	2.51	6,569.4	104.44
10 t ha^−1^	13.02 b	2.33	6,358.0	105.56
20 t ha^−1^	20.31 a	2.44	5,642.8	227.78
LSD (Tukey’s test)	3.32	NS	NS	NS
Nematode density (ND)				
Uninoculated	13.88	1.00 c	–	–
Low (50 J2)	12.62	2.69 b	665.22 b	115.00 b
High (500 J2)	12.05	4.02 a	13,126.0 a	420.56 a
LSD (Tukey’s test)	NS	0.214	1,596.2	91.71
*F-test values*				
Type of vermicompost (VC)	15.87^***^	22.52^***^	4.42^**^	29.10^***^
Rate of vermicompost (VCR)	32.06^**^	1.61^NS^	0.37^NS^	2.41^NS^
Nematode density (ND)	1.46^NS^	570.5^***^	245.36^***^	44.69^***^
VC × VCR	4.60^**^	15.89^***^	3.32^*^	2.02^NS^
VC × ND	2.78^*^	9.83^***^	3.37^*^	3.84^*^
VCR × ND	0.95^NS^	0.59^NS^	0.37^NS^	2.11^NS^
VC × VCR × ND	2.90^**^	5.88^***^	3.19^*^	1.92^NS^
**Root MSE ±**	4.65	0.38	3,375.06	193.92
**CV (%)**	36.20	14.80	48.94	72.41

Mean values followed by the same letter(s) within a column and treatment type are not significantly different at *p ≤* 0.05 using Tukey’s test (^NS^ *p* > 0.05; ^*^ *p* < 0.05; ^**^ *p* < 0.001; ^***^ *p* < 0.0001).

### 2.6 Statistical analysis

*In vitro* data were subjected to one-way ANOVA, a three-way ANOVA model was conducted for factorial data from the pot experiment using the PROC GLM procedure in SAS statistical software version 9.40 (SAS, 2004). Assumptions of ANOVA, including normality, homogeneity, and independence of experimental error, were tested before data analysis. All main and interaction effects between the three factors (type of vermicompost, rate, and nematode density) were determined using *F*-tests, and means were separated using Tukey’s honestly significant difference (HSD) test at *p* < 0.05. Mean comparisons for the three-way interaction effects were performed using the SAS PROC MIXED procedure, which facilitates computation of the LSD. However, for the field experiment, the LSD procedure (*p* < 0.05) was used following ANOVA in R.

## 3 Results

### 3.1 *In vitro* experiment

**Figure 1:**
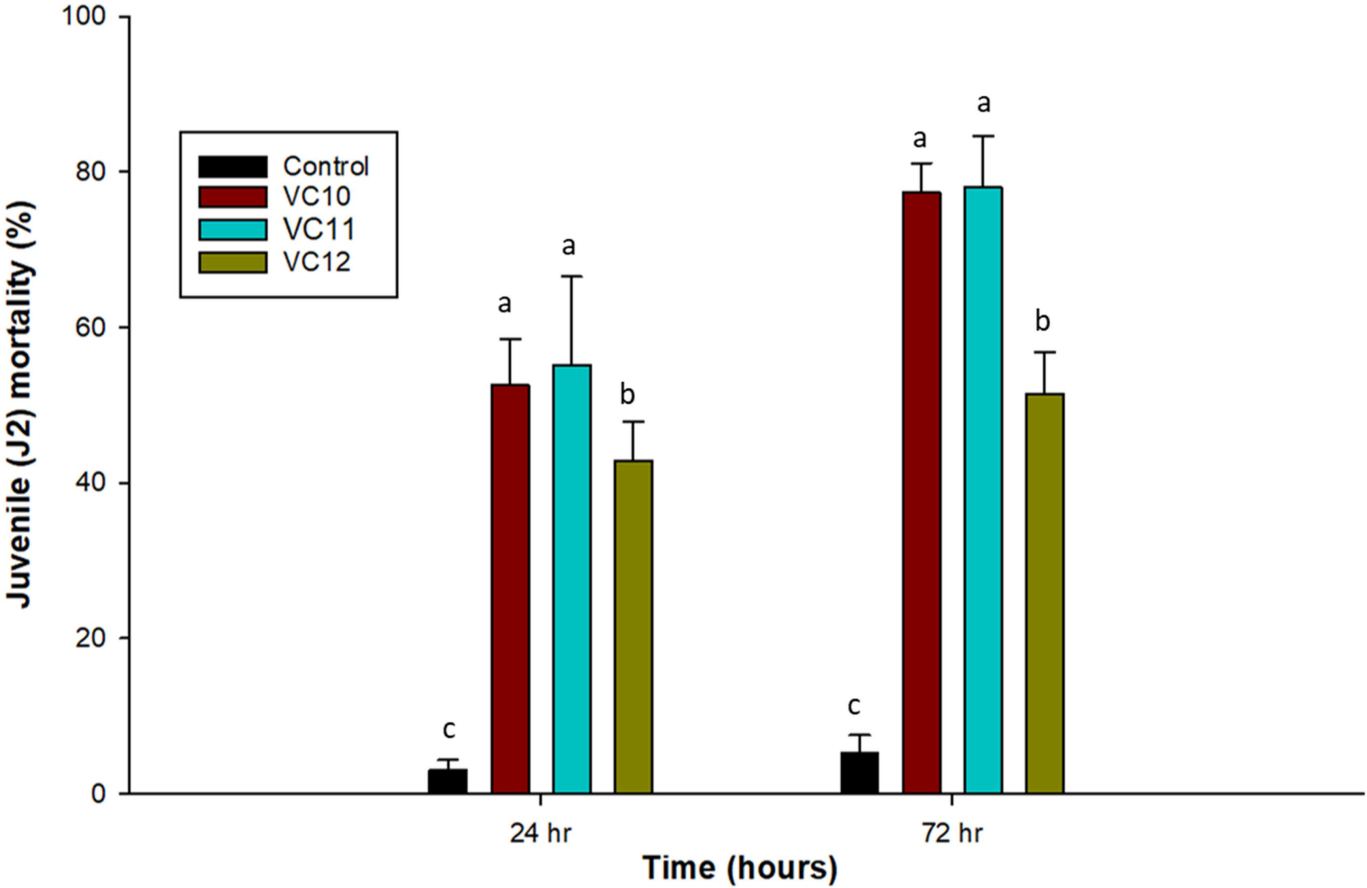
Effect of extracts from different vermicomposts (1:5 dilution) on mortality (%) of J2 of *M. incognita* at 24 (LSD = 10.82) and 72 h (LSD = 9.65) under in vitro conditions (*n* =5). Different letters (a–c) indicate significant differences among vermicompost extracts. Values represent means, and bars indicate means ± SE (*n* = 5).

A significant reduction in the number of second-stage J2 of *M. incognita* was observed after the direct application of the different vermicompost extracts. Vermicompost extracts have a nematoxic effect on J2 of RKNs, as evidenced by their higher mortality, where more than 50% were dead at 24 h and 78% after 72 h of exposure in both VC10 and VC11, compared to the control (**Figure 1**). Mortality was significantly higher in both VC10 and VC11 extracts than in VC12, which increased J2 mortality by only 43% and 51% after 24 and 72 h of exposure, respectively. Overall, there was a gradual increase in the mortality of J2 of *M. incognita* with increasing exposure time.

### 3.2 Pot experiment

Root fresh weight, root gall score, and J2 density in the soil sample were significantly (*p* < 0.05) affected by the main effect of vermicompost type (amended and unamended control). The main effect of vermicompost rate influenced only root fresh weight. Nematode density significantly affected root gall score and J2 populations in both root and soil samples. Factorial analysis of variance revealed significant effects of the main factors and their two-way interactions on nematode parameters, as well as a significant (*p* < 0.05) three-way interaction among vermicompost type (VC), vermicompost rate (VCR), and nematode density (ND) (VC × VCR × ND) (**Table 2**). This highlights the interaction between VC and its VCR with varying NDs, which significantly affected root fresh weight, gall score, and J2 density in roots. However, the three-factor interaction was not significant for J2 in the final soil sample, whereas the two-way interaction between vermicompost type and nematode density (VC × ND) had a significant effect on the soil J2 population (**Table 2**).

**Figure 2:**
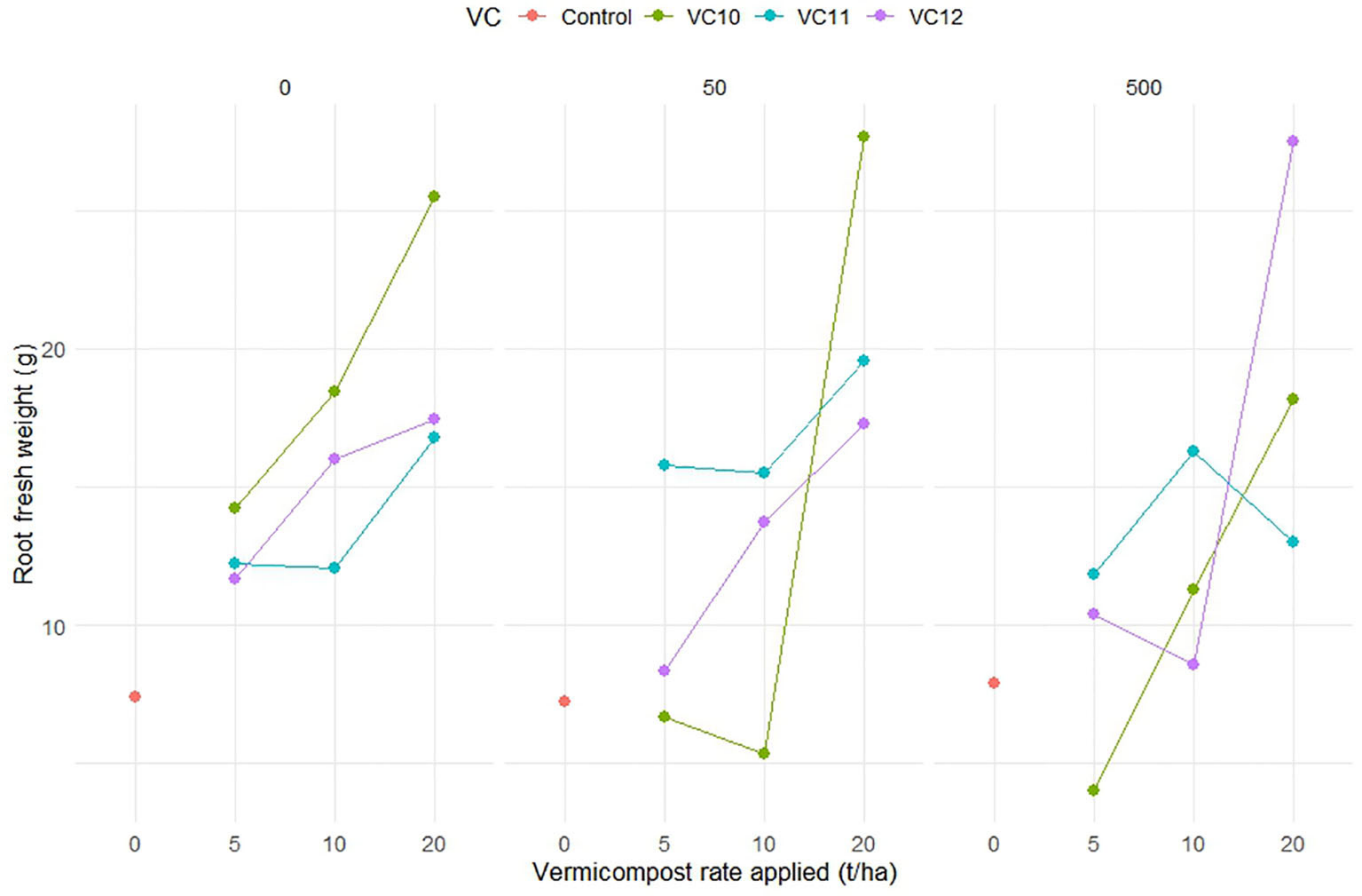
The interaction effect between type of vermicompost, rate applied and inoculated nematode density on root fresh weight (g) (LSD=7.718) of tomato plant under pot experiment.

Vermicompost amendments led to higher root fresh weight compared to the control, particularly in noninoculated plants and those inoculated with low nematode level. The highest root fresh weight was recorded at a high VC12 dose (20 t ha^−1^) under high nematode density. In contrast, the unamended but nematode-inoculated control exhibited lower root fresh weight. The highest root fresh weight was recorded when VC10 was applied at 20 t ha^−1^ in noninoculated plants (25.5 g) and at low ND (27.6 g). Similarly, a high application rate of VC12 (20 t ha^−1^) in plants inoculated with high ND resulted in a comparable root weight (27.4 g). However, in plants with high ND, lower application rates of VC12 (5 and 10 t ha^−1^) significantly reduced root weight, bringing it to levels similar to nonamended but inoculated treatments (**Figure 2**). Notably, when VC12 was applied at a high rate (20 t ha^−1^) under low ND conditions, root fresh weight decreased by 60% compared to VC10 applied at the same rate and nematode density.

**Figure 3:**
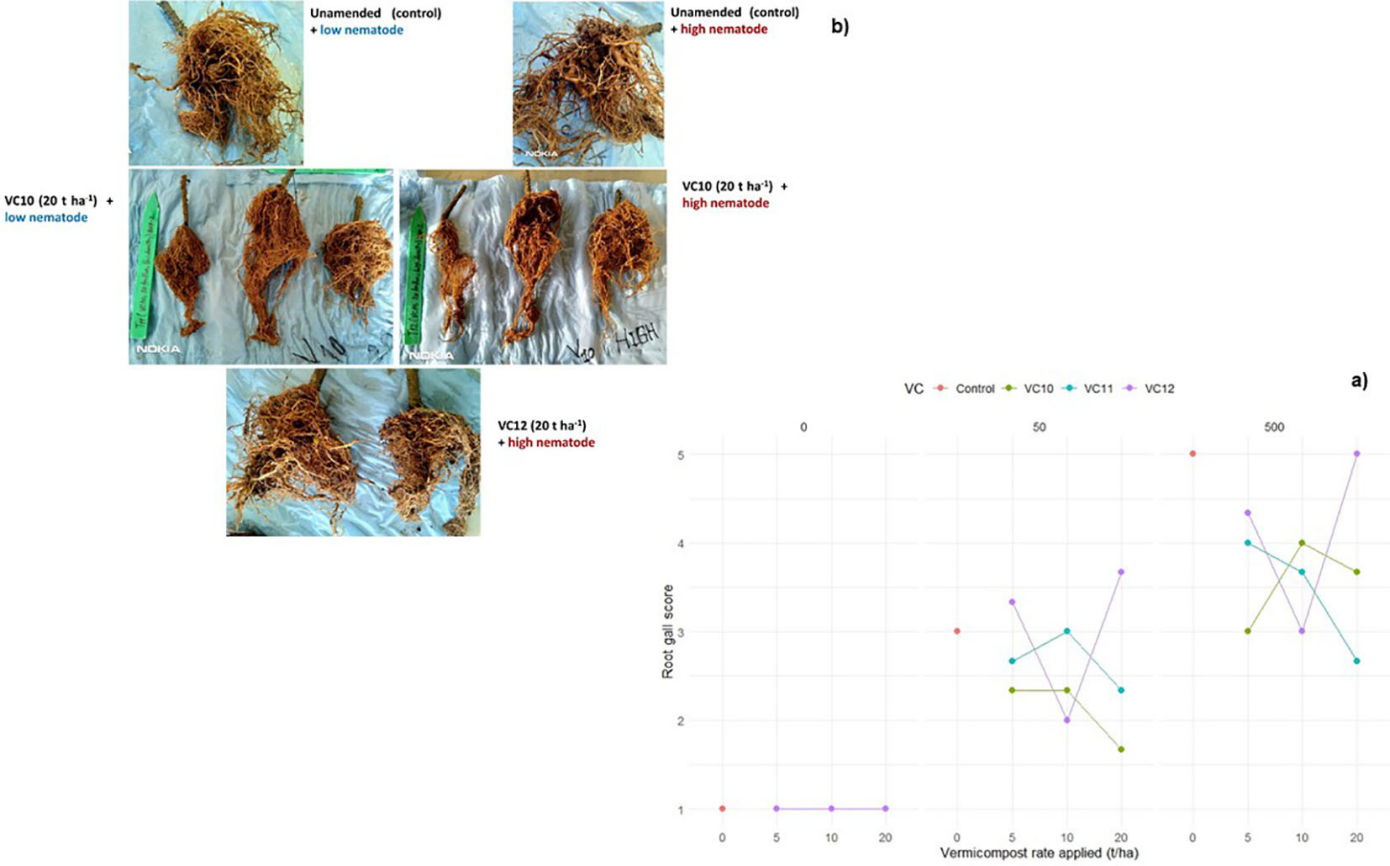
The interaction effect between type of vermicompost, rate applied and inoculated nematode density on gall score **(a)** of tomato roots (LSD=0.619) under pot experiment. Root gall structure of selected treatments **(b)**.

Tomato plants grown in nonamended soil with high nematode density inoculation had the highest number of root galls. VC12 applied at 20 t ha^−1^ under high nematode density resulted in the highest number of tomato root galls, but this was statistically comparable to the unamended soil at the same nematode density (**Figure 3**). In contrast, VC10 applied at 20 t ha^−1^ with low nematode density significantly reduced galls formation compared to the same application rate under high nematode density. Furthermore, VC10 applied at any rate (5, 10, or 20 t ha^−1^) under high nematode density resulted in fewer root galls than those observed in unamended but nematode-inoculated plants (**Figure 3**). VC11 also significantly reduced root gall formation, particularly at higher vermicompost application rates of 10 and 20 t ha^−1^.

**Figure 4:**
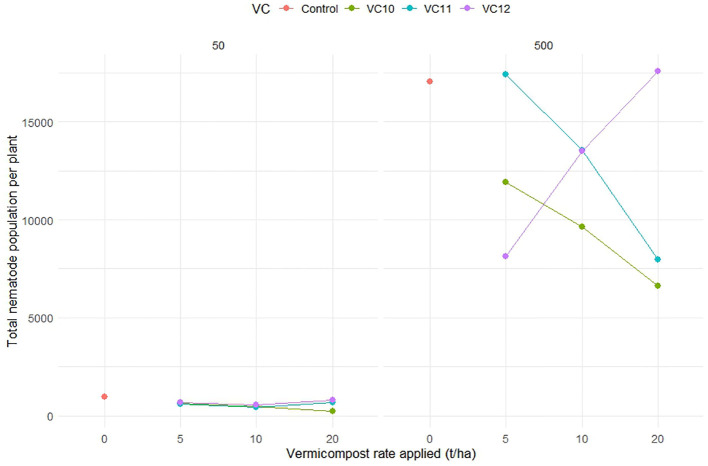
The interaction effect between type of vermicompost, rate applied and inoculated nematode density on *M. incognita* (egg + J2) of tomato roots (LSD=4375.68) under pot experiment.

The population of *M. incognita* (eggs + J2) per plant root system significantly decreased in both low (50 J2) and high (500 J2) nematode density treatments when VC10 and VC11 were applied at high doses (**Figure 4**). However, applying VC12 at high doses significantly increased the nematode population per root in tomato plants inoculated with a high nematode density. VC10 applied at 10 and 20 t ha^−1^ resulted in an average reduction of 11% and 250% in nematode population per root at low nematode density and 38% and 135% at high nematode density, respectively, compared to VC12 at doses of 10 and 20 t ha^−1^ under the same nematode densities.

**Figure 5:**
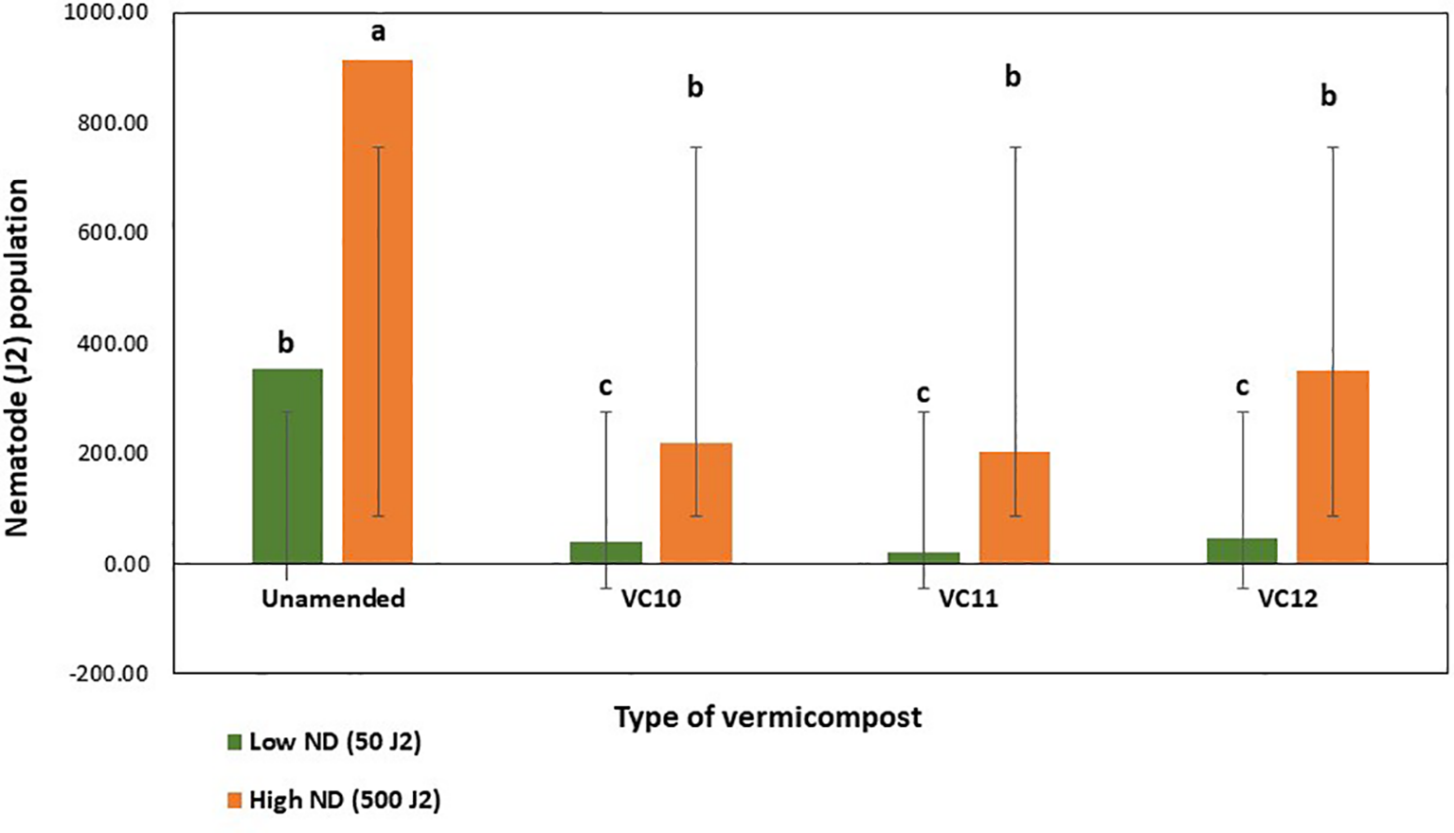
Effect of different types of vermicompost amendments on population of root knot nematode in soil inoculated at lower and higher density (LSD=200.05) under pot condition. Values show means and bars are means ± SE (*n*=3). Mean values followed by the same letter(s) are not significantly different at *P* ≤ 0.05 using Tukey’s test.

Analysis of the nematode population (J2) in the final soil sample showed that unamended soils at inoculation with a high nematode density had significantly higher J2 counts than those inoculated at a lower density (**Figure 5**). However, in vermicompost-amended soils, J2 populations were significantly reduced, with virtually no J2 recovered from the final soil samples, regardless of the type of vermicompost used as amendment.

The regression analysis, as depicted in **Supplementary Figure S1**, showed a weak negative relationship between J2 and root fresh weight, which was not statistically significant, indicating insufficient evidence to conclude that nematode density affects root fresh weight. In contrast, the analysis demonstrated a strong and statistically significant positive relationship between J2 and gall score.

### 3.3 Soil and vermicompost properties for the field experiment

The J2 population estimated using repeated extraction (the Baermann funnel technique) before soil amendment ranged from 15 to 30 J2 per 100 cm^3^ of soil. The results obtained from soil analysis (**Table 3**) showed that the experimental field was slightly acidic with the average organic carbon (OC) and total nitrogen (TN) contents of 2.32 and 0.162%, respectively. The OC and TN contents fall in the moderate range in accordance with the ratings suggested by Tekalign et al. (1991). Whereas soil available P (Bray-II) was less than 20 ppm and was low in accordance with Horneck et al. (2011).

**Table 3:** Characteristics of the soil and vermicomposts used for the field experiment.

Properties	Soil	VC10	VC11	VC12
Clay (%)	37	–	–	–
Silt (%)	24	–	–	–
Sand (%)	39	–	–	–
Texture class	Clay loam	–	–	–
pH (H_2_O)	5.68 (0.08)	7.22 (0.06)	7.34 (0.04)	7.36 (0.04)
OC% (DM)	2.32 (0.05)	35.08 (1.64)	37.03 (1.48)	35.24 (1.32)
TN% (DM)	0.162 (0.01)	1.21 (0.10)	1.14 (0.08)	1.11 (0.10)
Av. P (mg kg^−1^)	18.64 (1.14)	–	–	–
K (mg kg^−1^)	558.2 (87.2)	–	–	–
CEC (cmol(+) kg^−1^ soil)	20.12 (1.26)	–	–	–
Av. S (mg kg^−1^)	4.78 (0.84)	–	–	–

Values represent the means of three samples, with standard deviation in parentheses.

### 3.4 Effect of vermicompost on growth and yield of pepper under field condition

Vermicompost application at different rates (5, 10, and 20 t ha^−1^) had a significant effect (*p* < 0.05) on hot pepper (*Capsicum annuum* L.) growth, fruit number, and marketable yield under field conditions (**Table 4**). Regardless of the vermicompost type, hot pepper plants grown in vermicompost-amended soil (particularly in VC10 at 10 and 20 t ha^−1^) exhibited greater height and pod numbers, with longer and wider fruits, compared to the stunted plants in the control, farmers’ practice, and recommended fertilizer treatments. In addition, in unamended plots, J2 infestation caused root damage and reduced growth parameters. Vermicompost application reduced root gall counts compared to plants grown under the control, farmers’ practice, and recommended fertilizer treatments, which had disease incidence of 33.3%, 38.0%, and 30.9%, respectively. Hot pepper plants grown in soil treated with VC10 produced significantly more pods and had the highest marketable yield (**Table 4**), particularly at amendment rates of 10 and 20 t ha^−1^. Even at a relatively low VC10 dose (5 t ha^−1^), pepper growth remained high, with marketable yields increasing significantly by 77.8%, 49.6%, and 27.0% compared to plants in the control, farmers’ practice, and recommended fertilizer treatments, respectively (**Table 4**). On the other hand, hot pepper plants grown in treatments such as farmers’ practice and recommended fertilizer were significantly shorter, had fewer pods, and had marketable yields which were similar to plants in the control. Although vermicompost application increased marketable yields, nonmarketable fruit yields did not differ significantly between treatments.

**Table 4:** Effect of different treatments (vermicomposts at varying application rates, farmers’ practice, and the recommended fertilizer rate) on agronomic traits of hot pepper (*Capsicum annuum* L.) and soil chemical properties at the end of the field experiment.

Treatment	Plant height (cm)	No. fruits plant^−1^	Fruit width (cm)	Fruit length (cm)	Marketable yield (t ha^−1^)	Nonmarketable yield (t ha^−1^)	Disease incidence (%)	Root gall	pH	OC (%)	TN (%)	P (mg kg^−1^)	K (mg kg^−1^)	S (mg kg^−1^)	CEC (cmol(+) kg^−1^ of soil)
Control	51.2 g	19.3 f	1.70 e	7.50 b	1.22 g	0.363 bcde	33.33 ab	2.67 a	5.66 f	2.15 f	0.1433 e	18.11 g	534.6 d	4.99 e	18.56 g
VC10 at 5 t ha^−1^	62.7 bc	26.6 cd	2.09 c	14.38 a	2.17 cd	0.313 e	2.38 ef	1.33 c	5.72 de	2.55 ab	0.1690 ab	21.56 de	584.6 cd	9.27 c	23.96 de
VC10 at 10 t ha^−1^	69.3 a	36.3 b	2.38 a	14.80 a	2.93 a	0.351 cde	0.00 f	0.67 d	5.81 b	2.60 a	0.1730 a	24.61 ab	991.3 a	14.55 a	27.63 a
VC10 at 20 t ha^−1^	69.1 a	40.3 a	2.70 b	14.74 a	3.02 a	0.331 de	0.00 f	0.67 d	5.86 a	2.61 a	0.1733 a	25.79 a	978.0 a	15.20 a	27.41 ab
VC11 at 5 t ha^−1^	56.9 de	24.0 de	1.92 d	7.50 b	1.93 de	0.414 abcd	9.52 cd	1.67 b	5.71 de	2.38 e	0.1660 bc	20.16 ef	560.3 cd	6.72 d	20.41 f
VC11 at 10 t ha^−1^	59.7 cd	29.6 c	2.15 bc	14.39 a	2.31 bc	0.405 abcde	7.14 de	1.67 b	5.78 c	2.45 cd	0.1696 ab	21.65 de	776.6 b	10.07 c	24.60 d
VC11 at 20 t ha^−1^	62.3 bc	37.0 b	2.22 b	14.62 a	2.33 bc	0.406 abcd	7.14 de	1.33 b	5.81 b	2.51 bc	0.1733 a	22.68 cd	962.0 a	11.63 b	25.81 c
VC12 at 5 t ha^−1^	55.4 ef	24.3 de	1.87 d	14.71 a	1.71 ef	0.484 a	14.29 c	1.33 c	5.70 e	2.38 e	0.1630 c	19.35 fg	510.0 d	6.74 d	20.98 f
VC12 at 10 t ha^−1^	62.9 bc	29.3 c	2.14 bc	7.50 b	2.22 c	0.411 abcd	9.52 cd	1.33 c	5.73 d	2.43 de	0.1666 bc	21.52 de	893.3 a	11.24 b	23.33 e
VC12 at 20 t ha^−1^	64.4 b	35.0 b	2.08 c	14.41 a	2.53 b	0.365 bcde	7.14 de	1.33 c	5.77 c	2.49 bcd	0.1733 a	23.41 bc	1001.0 a	11.63 b	26.41 bc
Farmers practice (FP)	52.4 fg	20.3 f	1.73 e	14.60 a	1.45 fg	0.449 ab	38.09 a	2.67 a	5.67 f	2.11 f	0.1576 d	17.74 g	663.3 bc	5.60 e	18.73 g
Recom. fertilizer (RF)	52.1 fg	21.3 ef	1.76 e	14.64 a	1.71 ef	0.434 abc	30.95 b	2.67 a	5.66 f	2.00 g	0.1563 d	15.98 h	522.0 d	5.90 de	17.02 h
**LSD**	3.34	3.18	0.1078	0.444	0.281	0.093	6.14	0.300	0.023	0.067	0.0053	1.615	114.0	1.024	1.023
***F*-value**	30.50^***^	42.98^***^	41.30^***^	58.51^***^	32.50^***^	2.50^*^	40.08^***^	14.02^**^	67.30^***^	76.24^***^	25.53^***^	27.77^***^	28.18^***^	101.3^***^	110.3^***^

Mean values followed by the same letter(s) within a column are not significantly different at *p ≤* 0.05 using Tukey’s test (^NS^ *p* > 0.05; ^*^ *p* < 0.05; ^**^ *p* < 0.001; ^***^ *p* < 0.0001).

### 3.5 Effect of vermicompost on *M. incognita* population under field condition

**Figure 6:**
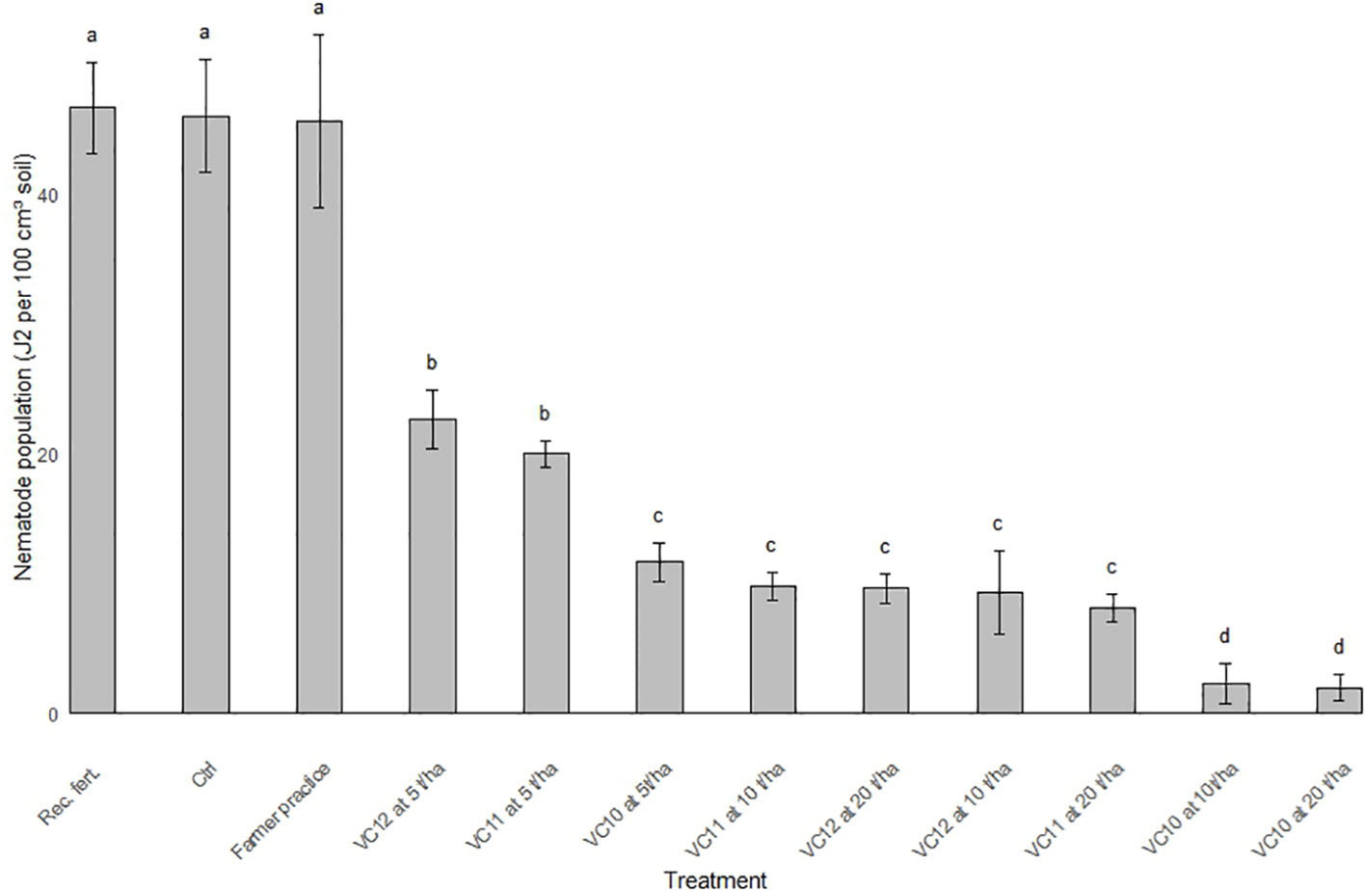
Nematode population (J2 100 cm^3^ soil^-1^) in the field soil as affected by different vermicomposts applied with different rates compared with farmers practice, recommended amount of fertilizer applied and control (LSD= 4.22). The bars subscribed by the same letter(s) within a treatment combination are not significantly different at *P* ≤ 0.05.

The results on J2 population density in the field soil, assessed as posttreatment populations following the application of vermicomposts, are shown in **Figure 6**. The figure illustrates the effects of different vermicompost treatments at varying application rates on J2 per 100 cm^3^ of soil compared to farmers’ practices, recommended fertilizer rate, and control. The control, farmers’ practice, and the recommended fertilizer showed the highest J2 population, with values significantly higher than any vermicompost treatment. The application of vermicomposts tested at 5 t ha^−1^ significantly reduced the nematode population compared to the control, FP, and recommended fertilizer, but they still resulted in a moderately high J2 population. Increasing the application rates of both VC10 (10 t ha^−1^ and 20 t ha^−1^) caused a further reduction in the J2 population. VC10 generally demonstrated superior nematode suppression compared to VC11 and VC12 at equivalent rates. For instance, VC10 at 10 and 20 t ha^−1^ performed better than VC11 and VC12 at the same rate and resulted in the most pronounced reduction (by above 75%) in J2 populations.

**Figure 7:**
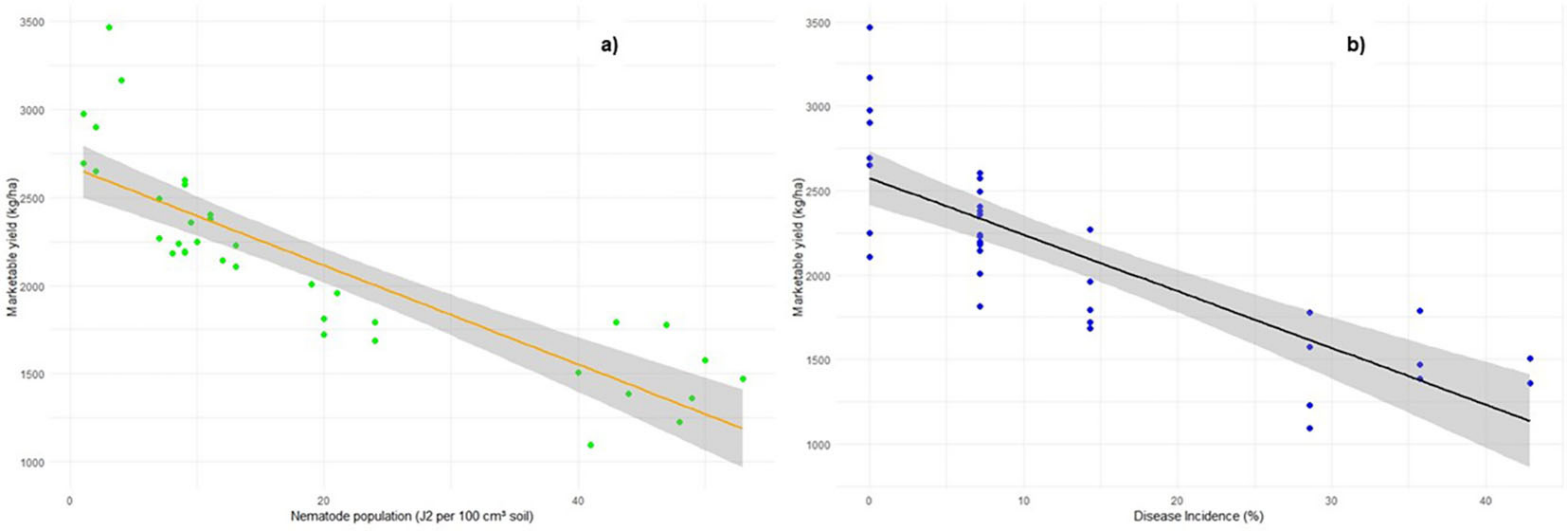
Relationship between marketable yield and **(a)** disease incidence and **(b)** nematode population, represented through regression analysis.

The regression analysis results, as shown in **Figure 7**, illustrate the relationship between marketable yield and disease incidence, as well as marketable yield and J2 population. The estimated intercept of 2,574.6 kg ha^−1^ represents the marketable yield in the absence of disease incidence, indicating the baseline yield. The regression plot reveals a highly significant downward trend, demonstrating a strong negative relationship between disease incidence and marketable yield (**Figure 7A**). The slope of − 33.54 indicates that for every 1% increase in disease incidence, the marketable yield decreases by approximately 33.54 kg ha^−1^. Similarly, a strong negative effect of the J2 population on pepper yield was observed. The slope suggests that for every unit increase in the J2 population, the marketable yield decreases by approximately 28.05 kg ha^−1^ (**Figure 7B**). Furthermore, the analysis showed that the J2 population explains more variation in marketable yield compared to disease incidence.

### 3.6 Effect of vermicompost on soil chemical properties after harvesting

Vermicompost application had significant effects (*p* < 0.001) on soil chemical properties (**Table 4**). Soils amended with vermicompost had significantly higher pH, OC, TN, P, K, S, and CEC values than the nonamended soils and the soils in farmers’ practice and treated with the recommended rate of fertilizer. Soil organic carbon, total N, and CEC contents increased with increasing vermicompost doses, but no significant difference was found between the vermicompost rates of 5, 10, and 20 t ha^−1^, especially for the VC10-amended soils for most of the soil properties. The highest OC content was found in the VC10 application at the rate of 20 t ha^−1^ (22% higher than the control soil), followed by the same vermicompost with the application rates of 5 and 10 t ha^−1^. Total N content also followed the same trend as OC. VC11 and VC12 treatments at the highest rate of 20 t ha^−1^ also increased the OC and total soil N contents, while the lowest contents were obtained in the control treatment and in soils under farmers’ practice and those treated with the recommended rate of fertilizer. The highest available *p*-values were observed at the highest rates (10 and 20 t ha^−1^) of all applied vermicomposts, and the same trend was observed for available K and S. in contrast, the lowest available P, K, and S contents were found in the control, farmers’ practice, and recommended fertilizer rate treatments. Vermicompost treatments also had significant effects on soil CEC. The greatest increase was recorded in the VC10 treatment at a rate of 20 t ha^−1^ (27.6 cmol(+) kg^−1^ soil), representing a 60% increase over the lowest CEC observed in soils treated with the recommended fertilizer rate (17.0 cmol(+) kg^−1^ soil) (**Table 4**). Overall, soil nutrient availability increased with higher vermicompost doses in amended treatments.

## 4 Discussion

### 4.1 Suppression of *M. incognita* J2s in roots using vermicompost under *in vitro* and pot conditions

Coyne et al. (2018) identified RKNs as one of the major biotic challenges to vegetable production, especially tomatoes, in Sub-Saharan Africa, significantly impacting food security in the region. Specifically, *M. incognita* is the most prevalent species in Ethiopian tomato-growing fields (Seid et al., 2019), highlighting the urgent need for sustainable RKN management strategies to reduce dependence on chemical nematicides. Given this need, vermicomposting emerges as a promising alternative. It is gaining attention as an affordable and ecologically sustainable method for valorizing organic waste into nutrient-rich and microbially active vermicompost, which can help control RKNs while protecting ecosystems (Bouchtaoui et al., 2024). The nematicidal effect of vermicompost used as an amendment or extract has been proven as an efficient biological control agent (Rostami et al., 2023). Results from the in vitro experiment showed a significant reduction in the number of J2 *M. incognita* when exposed to various vermicompost extracts after 24 and 72 h. These findings support the hypothesis that vermicompost extracts possess direct suppressive or high antagonistic activity against *M. incognita*. The observed effect on J2 mortality suggests that antagonistic microbes present in vermicompost extract produce toxic compounds that are detrimental to parasitic nematodes (Xiao et al., 2016; Kaur et al., 2022). In addition, vermicompost contains a substantial amount of N-containing compounds such as ammonia, which have been shown to have a nematicidal effect on PPN (Su et al., 2015). In the present study, a single high concentration of vermicompost extract (1:5) was used, and its effects on *M. incognita* mortality were evaluated over time. Consistent with our findings, Tikoria et al. (2022) reported that the mortality of J2 increased with higher concentrations of vermicompost extract and prolonged exposure durations. The increased mortality rate of *M. incognita* J2s observed in our study may be attributed to the relatively high concentration employed (1:5), as compared to previous studies, which commonly used compost-to-water ratios ranging from 1:3 to 1:10 for disease suppression (Arancon et al., 2007; Radovich et al., 2011). Similar to our study, Rao et al. (2017) reported an increase in mortality of *M. incognita* J2s with vermicompost extract. The enhanced microbial community and proliferation of beneficial bacteria during vermicomposting (Pathma and Sakthivel, 2012; Arancon et al., 2012) likely contributed to the observed nematicidal activity. The observed nematicidal effects in the in vitro experiment can be attributed to biochemical metabolites retained in the cell-free filtrates, which are likely produced by microbial communities present in the vermicomposts. Although microbial activity was not directly analyzed in the current study, previous work by Gebrehana et al. (2023) established the presence of diverse microbial populations and their metabolic activity in these vermicomposts. Future studies should aim to directly analyze microbial contributions to nematode suppression by isolating and testing specific microbial strains or their metabolites. Bhat et al. (2023) also reported that biocontrol agents like vermicompost extracts consist of a wide range of organisms, such as bacteria, fungi, viruses, and protozoans, which naturally act as parasitic nematode antagonists. This is supported by the recent findings of Liang et al. (2024), who screened antagonistic microbes (bacteria isolates) against *M. incognita* from fresh vermicompost to assess their biocontrol potential in tomato and cucumber crops. Furthermore, Rostami et al. (2021) also isolated antagonistic bacteria from liquid vermicompost that exhibited activity against *M. javanica* RKNs.

**Figure 8:**
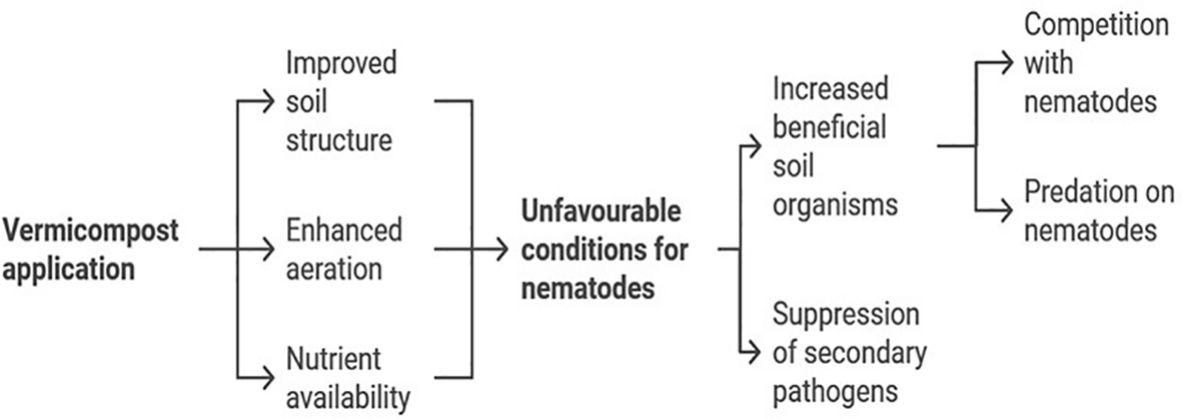
Mechanisms of nematicidal activity in vermicompost.

We found consistent results across our experiments, as the findings from the *in vitro* exposure experiment were validated by the pot assay. Our pot experiment demonstrated that analyzing J2 populations in relation to root weight across different treatments revealed that increasing doses of vermicompost reduced the rate of J2 population build-up, particularly in VC10 and VC11. In contrast, VC12 led to an increased J2 population and gall score with higher vermicompost rates when inoculated with a high nematode density. Similarly, Tabarant et al. (2011) reported that the suppression of plant-parasitic nematodes varied depending on the nature of the amendments used. VC10 produced from a substrate consisting of cow manure mixed with soybean and banana residues using *Eudrilus eugeniae*, significantly reduced the population of J2 in tomato roots, with suppression increasing at higher application rates. The notable suppression observed with VC10 could be attributed to its initially higher total nitrogen concentration, which may release large amounts of ammonia known to be toxic to nematodes (Oka, 2010). Similar findings by Nath et al. (2011) and Rostami et al. (2014) reported reductions in*M. incognita* populations following vermicompost application. This reduction may be linked to the promotion of root defense against RKNs through the accumulation of defense compounds facilitated by vermicompost (Xiao et al., 2016). Although the exact mechanisms underlying disease suppression remain unclear, studies suggest several possibilities (Oka, 2010; McSorley, 2011; Pathma and Sakthivel, 2012; Rosskopf et al., 2020). These mechanisms include the release of nematicidal compounds (e.g., ammonia), stimulation of antagonistic microbes, and improved plant vigor and tolerance to RKNs (Simsek-Ersahin, 2011). A combination of these mechanisms likely contributes to nematode suppression in vermicompost-amended soils. Vermicompost, the product of organic waste decomposition by earthworms and associated microorganisms, exhibits nematicidal activity through microbial, biochemical, and physical mechanisms. These mechanisms disrupt the life cycle of root-knot nematodes (*Meloidogyne* spp.) and other plant-parasitic nematodes, reducing their populations and suppressing infestations. The detailed mechanisms of vermicompost and its derivatives (e.g., vermicompost tea) have been recently reviewed (Rehman et al., 2023; Bouchtaoui et al., 2024). **Figure 8** presents a schematic representation of the proposed mechanisms underlying vermicompost’s nematicidal activity, including (1) microbial antagonists, (2) biochemical metabolites responsible for nematode suppression, and (3) indirect mechanisms such as enhanced plant resistance through improved growth parameters (Zuhair et al., 2022; Bouchtaoui et al., 2024). Zuhair et al. (2022) demonstrated that vermicompost derived from different plant waste origins effectively controlled RKNs in infected tomato plants. This suggests that a combination of the mechanisms illustrated in **Figure 8** contributes to the efficacy of vermicompost in nematode suppression.

Overall, our laboratory and pot studies revealed that the application of VC10 and VC11 at high rates (10 and 20 t ha^−1^) showed promising results in mitigating *M. incognita* populations and could be beneficial in alleviating the nematode damage in tomato plants. In addition, the regression analysis used shows no significant relationship between the J2 population and root fresh weight. The model explains almost no variability in root fresh weight, suggesting nematode density is not a good predictor for root fresh weight. The results suggest that while J2s have a limited effect on reducing root fresh weight, they strongly influence the severity of root galling, as reflected by the increasing gall score (**Supplementary Figure S1**). These findings highlight the differential impacts of nematode density on plant health, where gall formation is a more sensitive indicator of nematode pressure than root weight.

### 4.2 Vermicompost amendment effects on soil quality and yield of hot pepper under field condition

Our field experiment provided valuable insights into the impact of vermicompost amendments on RKNs suppression and hot pepper yield. The results demonstrated that the effectiveness of vermicompost varied depending on the type and application rate. Comparisons among plots treated with vermicompost, inorganic fertilizer, farmers’ practice, and the control revealed significant improvements in several growth parameters. Plant height, number of pods per plant, and fruit size were all higher in vermicompost-treated plots. One of the contributing factors to these observed growth increments was the abundant N supply and microbial biomass in vermicomposts, which positively influenced plant growth in farmers’ fields and led to a notable reduction in root gall formation. This effect is likely attributed to the increase in microbial biomass facilitated by vermicompost application. Previous studies by Arancon et al. (2002, 2003) have also highlighted the ability of vermicompost to enhance microbial competition and suppress plant-parasitic nematodes. Furthermore, the application of vermicompost may induce resistance in pepper plants due to the presence of antibiotics and actinomycetes (Simsek-Ersahin, 2011). Additionally, Xiao et al. (2016) and Kaur et al. (2022) reported that vermicompost amendments enhanced RKN suppressiveness.

Vermicompost amendments applied at different rates not only improved the growth and fruiting of hot pepper plants but also significantly increased marketable yield. The highest marketable yields were obtained in VC10 at high application rates of 10 and 20 t ha^−1^, exceeding those of the control, farmer practices, and recommended fertilizer treatments by more than 90% for both rates (**Figure 6**). Several studies have reported a decrease in nematode populations alongside improved plant growth and yield parameters with higher vermicompost doses (Rostami et al., 2014; Xiao et al., 2016). Hot pepper yield exhibited a progressive increase with higher vermicompost application rates, although the magnitude of these increments varied depending on the vermicompost type. Vermicompost harbors diverse microbial communities, and its application to the soil can stimulate microbial activity, potentially exerting a direct influence on plant growth and yield. Nath et al. (2011) observed a reduction in the nematode population (*M. incognita*) and a higher marketable fruit yield in plots enriched with vermicompost compared to the control. Similarly, Arancon et al. (2003) reported improved growth and marketable fruit yields in field-grown tomatoes, peppers, and strawberries following vermicompost amendments. These positive effects on plant growth and fruit yield can be attributed, at least in part, to the substantial increase in soil microbial biomass after vermicompost application. This increase triggers the production of growth-regulating hormones, such as indole acetic acid, gibberellins, cytokinins, and humic acids (Edwards et al., 2004; Arancon et al., 2012). In addition to its positive influence on healthy plant growth, vermicompost application also contributed to the improvement of soil chemical properties. Our results revealed that vermicompost significantly increased soil pH, organic carbon, CEC, and nutrient availability. These improvements were more pronounced at higher vermicompost application rates, surpassing both farmer practices and the recommended rate of fertilizer. The increase in soil pH suggests that vermicompost has neutralizing effect on soil acidity, thereby enhancing the availability of N and P in vermicompost-amended soils compared to chemical fertilizers and farmer practices. The observed increase in CEC and SOC highlights the ability of vermicompost to enhance soil buffering capacity and ameliorate acidity in Nitisols. These changes in the chemical properties of amended soils suggest a significant impact on soil biological properties, including increased microbial biomass and activity (Lazcano and Domínguez, 2011). Similarly, Tejada and Gonzalez (2009) reported that vermicompost application increases nutrient availability, CEC, and soil water-holding capacity. In addition, changes in soil properties (e.g., pH, organic matter content, and nutrient availability) and alterations in soil microbial communities are key mechanisms by which vermicompost suppresses RKNs (Bouchtaoui et al., 2024). Therefore, the application of vermicompost in farmers’ fields holds the potential to markedly improve soil quality by increasing soil microbial populations, all of which have positive effects on plant growth and development (Arancon et al., 2003). The results of the present study suggest that vermicompost application is a sustainable agricultural practice for smallholder farmers, effectively reducing economic losses caused by *M. incognita* while simultaneously improving soil quality and the yield of tomato and hot pepper plants. Recently, Walia and Kaur (2024) also reported that vegetable and fruit crops benefit significantly from vermicompost application due to overall improved soil health and enhanced nutrient availability. This effect may be attributed to its organic matter content, which improved soil structure, water-holding capacity, and microbial diversity. By enhancing these factors, vermicompost promotes robust root development and increased vegetable and fruit production (Walia and Kaur, 2024).

### 4.3 Effect of vermicompost amendment on J2 population density in hot pepper under field conditions

The posttreatment nematode population density was assessed using the same methodology as the initial population evaluation. After harvesting, soil samples were processed using the modified Baermann funnel technique to extract J2, which were then counted under a stereomicroscope. The posttreatment J2 population densities were compared to the initial population to evaluate treatment effectiveness. This comparison provided insights into the suppressive effects of the amendments on *M. incognita*. Vermicompost treatments resulted in a significantly greater reduction in J2 than the untreated (control) and conventional treatments, which tended to increase J2 populations (**Figure 6**). The observed increase in J2 (*M. incognita*) populations in conventional treatments, compared to organic amendments such as vermicompost, may be attributed to several potential mechanisms. Conventional chemical fertilizers can reduce microbial diversity and abundance in the soil by altering pH and nutrient dynamics. This decline in beneficial microbial populations, including nematophagous fungi and bacteria, can lower biological control agents that naturally suppress nematodes. For instance, bacteria in the genera *Bacillus* and *Pseudomonas*, as well as fungi like *Paecilomyces lilacinus*, are known to antagonize nematodes. Their reduced presence may contribute to an unchecked increase in nematode populations (Khan et al., 2006). Unlike organic amendments such as vermicompost, which contain toxic metabolites like phenolic compounds, humic acids, and microbial by-products that suppress nematodes, chemical fertilizers lack these bioactive components. Organic toxic compounds have been shown to directly inhibit nematode hatching and activity (Edwards et al., 2011). The absence of these suppressive agents in chemical treatments may facilitate nematode proliferation. In addition, repeated use of chemical fertilizers may inadvertently select nematode populations that are more resilient to adverse soil conditions, leading to an increased prevalence of nematodes that thrive under such treatments. The results showed that among all the tested vermicomposts, VC10 applied at higher rates (10 and 20 t ha^−1^) was more effective in reducing J2 populations by several folds compared to conventional treatments (control, FP, and recommended fertilizer) under field conditions. The data suggest a dose-dependent effect, where higher vermicompost application rates led to greater nematode suppression. This finding highlights the potential of vermicompost as an effective and sustainable strategy for nematode management in agricultural soils, with VC10 at higher rates demonstrating the greatest efficacy.

In addition, a significantly strong negative relationship between disease incidence and marketable yield was observed in the present study, indicating that disease incidence had the greatest influence on pepper yield. This could be attributed to nematode-infected roots deteriorating, which compromises host health, increases pest and disease pressure, and leads to greater reliance on pesticides (Coyne et al., 2018). Similarly, nematode density exhibited a strong negative effect on marketable yield. These results suggest that maximizing pepper yield can be achieved by suppressing J2 population density in field soil, thereby reducing disease incidence and minimizing yield loss.

### 4.4 Marketable yield as influenced by nutrient supply from vermicompost

The improved marketable yield of pepper resulting from vermicompost amendments may be attributed to enhanced bioavailability of nutrients (N, P, K, and S) and an increase in soil pH. This rise in soil pH is particularly important in the study area, where the soil is highly weathered and has a low pH, which can affect P availability and hinder pepper growth. In both pot and field experiments, we applied the same rates of vermicompost, primarily aiming to suppress PPN. Although vermicompost amendments outperformed treatments such as the recommended rate of chemical fertilizer and farmers’ practice, trends within treatments were not consistent due to variations in vermicompost type and application rates. While the rates of vermicompost may seem high compared to farmers’ practice (3 t ha^−1^ cow manure + 23 kg N ha^−1^), it should be noted that approximately one-fourth of the recommended N (23 kg N ha^−1^) is also supplied by the chemical fertilizer in this treatment. This suggests that vermicompost application may have stimulated additional mechanisms that enhance the efficiency of N uptake. This effect was particularly observed in VC10, where pepper fruit yield significantly increased even at a lower application rate (5 t ha^−1^) compared to 20 t ha^−1^, suggesting a nonnutritional effect of vermicompost, such as stimulation of hormone production. The strong yield response of hot pepper to vermicompost application (especially at high rates) highlights its potential for efficiently supplying nitrogen. Agegnehu et al. (2016) reported that adequate N availability is crucial for optimal nitrogen uptake by plants, and organic amendments like vermicompost can help maintain this availability. Interestingly, applying only half the equivalent nitrogen from VC10 at 5 t ha^−1^ (approximately 60 kg N ha^−1^) appeared sufficient to sustain pepper growth and yield compared to the N supplied from chemical fertilizer, as evidenced by the N levels after harvest. Moreover, increasing the vermicompost application beyond 10 t ha^−1^ (i.e., 20 t ha^−1^) for VC10 and VC11 did not significantly enhance hot pepper yield, potentially due to decreased N use efficiency. Compared with chemical fertilization, vermicompost application significantly increased the marketable yield of hot pepper, indicating that vermicompost provides essential micro- and macronutrients (such as K and S) in addition to N and P, which could otherwise limit yield and agronomic benefits. This finding is supported by Kiyasudeen et al. (2015) and Sharma and Garg (2019), who reported that vermicompost is rich in micro- and macronutrients. Overall, vermicompost appears to be a viable alternative to chemical fertilizers, as it not only enhances plant growth but also improves soil quality (Jouquet et al., 2011). Therefore, vermicompost can serve as an effective nutrient management strategy to optimize crop growth while concurrently protecting plants from pests and diseases.

## 5 Conclusion

Our study, comprising both *in vitro* and pot experiments, demonstrates the beneficial effect of high-quality vermicompost (particularly VC10 and VC11) in suppressing root-knot nematode (*M. incognita*). It is important to note that the effectiveness of vermicompost in nematode suppression varied on the type of vermicompost used. The field experiment demonstrated that vermicompost application effectively controlled RKNs and significantly increased the yield of hot pepper. In summary, our results highlight the importance of considering both the type and rate of vermicompost application when aiming to suppress RKNs and improve hot pepper yield. While applying vermicompost at 10 t ha^−1^ increased pepper yield in the farmer’s field, no additional benefits were observed at a higher application rate of 20 t ha^−1^, particularly for VC10. This suggests that the interaction between vermicompost type and application rate can influence its effectiveness in nematode suppression. In addition to suppressing nematodes and increasing yield, vermicompost positively impacted soil chemical properties, primarily through its microbial activity. The study also found that even at a low application rate of 5 t ha^−1^, vermicompost effectively suppressed RKN infestation, improved the marketable yield of hot pepper, and enhanced soil chemical properties under field conditions.

## Data Availability

The raw data supporting the conclusions of this article will be made available by the authors, without undue reservation.
