# Supplementary material for: Suppression potential of selected vermicomposts against root-knot nematode (Meloidogyne incognita) under in vitro, pot, and field conditions
Source: Front Plant Sci. 2025 Mar 11;16:1532800. doi: 10.3389/fpls.2025.1532800 (PMC11932992; doi:10.3389/fpls.2025.1532800)
Supplement: Supplementary file 1 [file Image1.pdf]

Suppression potential of selected vermicomposts against root-knot nematode (*Meloidogyne incognita*) under in vitro, pot and field conditions

Zerihun Getachew Gebrehana[a,b,*], Mesfin T. Gebremikael[a,c], Sheleme Beyene[d], Wim M.L. Wesemael[e,f], Stefaan De Neve[a]

[a] Research Group Soil Fertility and Nutrient Management, Faculty of Bioscience Engineering, Ghent University, Coupure Links, 653, 9000 Ghent, Belgium
[b] Assosa Agricultural Research Center, Ethiopian Institute of Agriculture Research, P.O. Box 2003, Addis Ababa, Ethiopia
[c] Department of food science, Aarhus University, Agro Food Park 48, 8200 Aarhus, Denmark
[d] School of Plant and Horticultural Sciences, College of Agriculture, Hawassa University, Ethiopia
[e] Institute for Agricultural and Fisheries Research (ILVO), Burg Van Gansberghelaan 96, B-9820 Merelbeke, Belgium
[f] Laboratory for Agrozoology, Faculty of Bioscience Engineering, Ghent University, Coupure links 653, B-9000 Ghent, Belgium
**E-mail address:* Zerihun.GetachewGebrehana@UGent.be

Supplementary figure

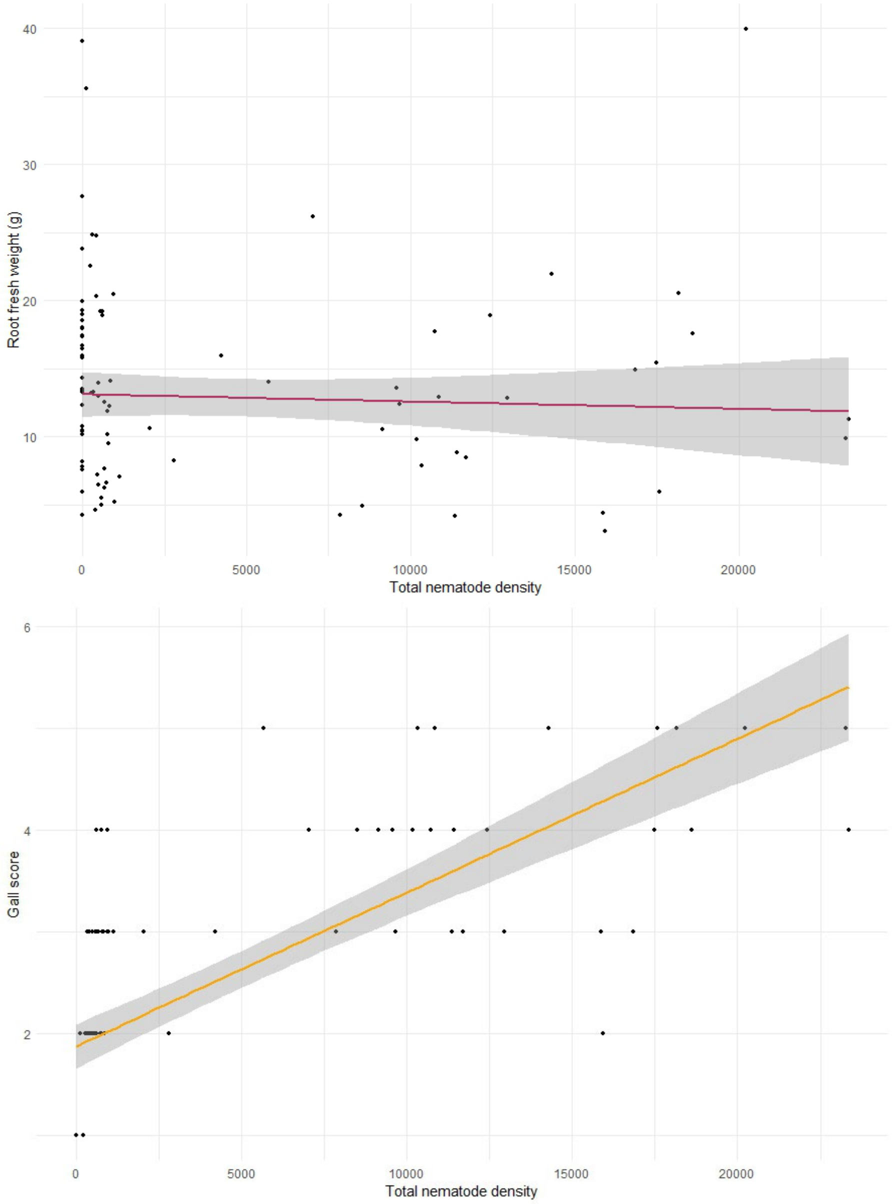

Fig S.1 Relationship between total nematode population and (a) root-fresh weight and (b) gall score, represented through regression analysis.
